# Pharmacological and Biophysical Characteristics of Picrotoxin-Resistant, δSubunit-Containing GABA_A_ Receptors

**DOI:** 10.3389/fnsyn.2021.763411

**Published:** 2021-11-18

**Authors:** Hong-Jin Shu, Xinguo Lu, John Bracamontes, Joe Henry Steinbach, Charles F. Zorumski, Steven Mennerick

**Affiliations:** ^1^Department of Psychiatry, Washington University in St. Louis, School of Medicine, St. Louis, MO, United States; ^2^Department of Anesthesiology, Washington University in St. Louis, School of Medicine, St. Louis, MO, United States; ^3^Taylor Family Institute for Innovative Psychiatric Research, Washington University in St. Louis, School of Medicine, St. Louis, MO, United States; ^4^Department of Neuroscience, Washington University in St. Louis, School of Medicine, St. Louis, MO, United States

**Keywords:** GABA allosteric modulators, inhibition, antidepressant, dentate gyrus, neurosteroid, ethanol

## Abstract

GABA_A_ receptors (GABA_A_Rs) play a crucial role in inhibition in the central nervous system. GABA_A_Rs containing the δ subunit mediate tonic inhibition, have distinctive pharmacological properties and are associated with disorders of the nervous system. To explore this receptor sub-class, we recently developed mice with δ-containing receptors rendered resistant to the common non-competitive antagonist picrotoxin (PTX). Resistance was achieved with a knock-in point mutation (T269Y; T6’Y) in the mouse genome. Here we characterize pharmacological and biophysical features of GABA_A_Rs containing the mutated subunit to contextualize results from the KI mice. Recombinant receptors containing δ T6’Y plus WT α4 and WT β2 subunits exhibited 3-fold lower EC_50_ values for GABA but not THIP. GABA EC_50_ values in native receptors containing the mutated subunit were in the low micromolar range, in contrast with some published results that have suggested nM sensitivity of recombinant receptors. Rectification properties of δ-containing GABA_A_Rs were similar to γ2-containing receptors. Receptors containing δ T6’Y had marginally weaker sensitivity to positive allosteric modulators, likely a secondary consequence of differing GABA sensitivity. Overexpression of δT6’Y in neurons resulted in robust PTX-insensitive IPSCs, suggesting that δ-containing receptors are readily recruited by synaptically released GABA. Overall, our results give context to the use of δ receptors with the T6’Y mutation to explore the roles of δ-containing receptors in inhibition.

## Introduction

Activation of GABA_A_Rs inhibits targets expressing the receptors and sculpts patterns of the activity responsible for thought, emotion, and in fact virtually all brain functions. Two major subclasses of GABA_A_Rs are prominently expressed in the CNS, sometimes within the same cell type. γ2-Containing receptors are typically found at synapses and mediate phasic inhibition caused by the synaptic release of GABA. In contrast, δ-containing receptors are expressed in more circumscribed populations of neurons and tend to mediate tonic currents to ambient GABA and a slow component of IPSCs (Nusser et al., 1998; Wei et al., 2003; Martenson et al., 2017; Sun et al., 2018, 2020). δ-Containing receptors are also thought to have high sensitivity to certain positive allosteric modulators, such as neurosteroids and ethanol (Mihalek et al., 1999; Spigelman et al., 2003; Stell et al., 2003; Wei et al., 2004; Glykys et al., 2007), although our recent results questioned this selectivity (Lu et al., 2020). Features of both GABA_A_R subclasses make them attractive targets for therapeutics in different situations. A more nuanced understanding of the properties of δ-containing and γ2-containing receptors would aid the search for targeted therapeutics and aid understanding of the specific roles of different receptor sub-classes.

To aid the exploration of the two subclasses of GABA_A_Rs, we recently introduced knock-in (KI) mouse lines that carry pharmacoresistance to picrotoxin (PTX) as a means of isolating the two receptor classes (Sun et al., 2018). Studies of the KI mice revealed a greater contribution of δ-containing receptors to IPSCs of dentate granule cells (DGCs) than expected (Sun et al., 2018, 2020). The KI mice also revealed little or no preferential sensitivity to neurosteroids (Lu et al., 2020). An open question is whether receptors containing the mutant δ subunit are sufficiently different pharmacologically from WT δ-containing receptors to explain the unexpected results.

PTX-resistant GABA_A_R subunits were engineered and partially characterized previously. It was found that the T6’Y mutation could be made in any subunit of a pentameric GABA_A_R to reduce or eliminate PTX sensitivity (Gurley et al., 1995; Sedelnikova et al., 2006; Erkkila et al., 2008). In γ2 receptors, this amino acid substitution had little effect on GABA EC_50_ (Erkkila et al., 2008), but when introduced into mice, γ2T6’Y causes a seizure phenotype that appears to arise from slightly altered kinetics of synaptic currents (Sun et al., 2019). The comparable mutation in δ subunit-containing receptors has not been characterized in as much detail, although we have shown that kinetics and agonist sensitivity are similar to WT in native DGCs (Sun et al., 2018, 2019).

Here we use a combination of recombinant receptor subunits, where we can directly compare WT and mutated subunits, and tissue slices, where subunits are expressed in a native environment, to examine pharmacological and biophysical properties of GABA_A_Rs that may be important to their physiological role. We found that receptors with a mutated δ subunit exhibit ~3-fold higher sensitivity to GABA, leading to marginally altered responsiveness to allosteric modulators. We also found that mutant receptors exhibit similar rectification to WT δ-containing receptors and γ2-containing receptors in native cells. Thus, rectification is unlikely to increase the prominence of δ-containing receptors relative to γ2-containing receptors at transmembrane voltages positive to rest. Overall, our results show that pharmacoresistant KI mice are valid tools for evaluating the roles of GABA_A_R sub-classes if caveats are considered.

## Methods

### Cell Culture and Recombinant Receptor Expression

The murine neuroblastoma Neuro-2a (N2a; ATCC No. CCL-131) cell line was grown in Dulbecco’s Modified Eagle’s medium (DMEM) supplemented with 10% (v/v) FBS, 2 mM glutamine plus 100 U/ml penicillin, and 0.1 mg/ml streptomycin in an atmosphere of 5% CO_2_ and 95% air, and was maintained at sub-confluent densities in the growth media.

GABA_A_Rs were expressed in N2a cells by transiently transfecting the cDNAs of free α4, β2, and δ subunits of the GABA_A_R. The cDNA for human α4 was obtained from Dr. Paul Whiting (Merck, Harlow, Essex, UK), rat β2 was obtained from Dr. David Weiss (University of Texas Health Science Center, San Antonio, TX, USA), and rat δ was provided by Dr. Robert Macdonald (Vanderbilt University, Nashville, TN, USA). The δ(T269Y; δT6’Y) mutation was used to confer resistance to PTX (Gurley et al., 1995). The mutations were made using the QuikChange site-directed mutagenesis kit (Agilent Technologies, Santa Clara, CA). The δ subunit contained the FLAG epitope in the amino terminus of the subunit (Ueno et al., 1996). Transfection was carried out using a total of 1.2 μg of cDNA in the ratio of 1:1:2(α:β:δ), along with reporter plasmid (GFP, Clontech Laboratories, Mountain View, CA) as a positive transfection marker. Lipofectamine2000 (Life Technologies, Carlsbad, CA) was used as a transfection reagent according to the manufacture’s protocol. Electrophysiological experiments were performed 48–72 h later following transfection.

Rat primary cultures of hippocampal cells were prepared from 1 to 3 day postnatal Sprague Dawley rats, as described previously (Mennerick et al., 1995). Tissue was prepared according to protocols approved by the Institutional Animal Care and Use Committee. The GABA_A_R subunits were transduced with AAV8-Syn-α4-IRES-GFP, AAV8-Syn-δT6’Y-IRES-GFP, and AAV8-Syn-GFP virus 5 days after plating (Syn refers to the human synapsin promoter). Viral preparation was supported by the Hope Center Viral Vectors Core at Washington University School of Medicine. Electrophysiological experiments were performed 7 days following viral infection.

### Whole-Cell Patch-Clamp Recording in Cell Cultures

Experiments on N2a cells were conducted using standard whole-cell techniques. Non-transfected cells had no response to GABA. The bath solution containing (in mM): 138 NaCl, 4 KCl, 2 CaCl_2_, 1 MgCl_2_, 10 glucose, and 10 HEPES; pH 7.25. Patch pipettes were filled with an internal solution containing (in mM): 130 CsCl, 4 NaCl, 4 MgCl_2_, 0.5 CaCl_2_, 5 EGTA, 10 HEPES, pH 7.25. When filled with this solution, pipette tip resistance was 3–6 MΩ. Cells were clamped at −70 mV. Drugs and elevated potassium were applied with a multibarrel, gravity-driven local perfusion system. The estimated solution exchange times were 10–20 ms, measured by the change in junction currents at the tip of an open patch pipette. A typical drug application consisted of recording 2 s of baseline, followed by a 4–8 s drug application and a bath application (up to 1 min) until full recovery. Culture recordings were performed at room temperature.

Currents were filtered at 2 kHz and recorded at 5 kHz with an Axopatch 200 B amplifier (Molecular Devices, San Jose, CA). The analysis of current traces aimed at determining the peak current was conducted using pClamp 9.0 software (Molecular Devices). All recordings were performed at room temperature. Charge calculations for phasic currents were performed in Clampfit with cursors set at the onset and of K^+^ application and using the built-in statistics functions of Clampfit to calculate the integral of current over time.

EC_50_ values for GABA and THIP were derived from fits to the Hill equation with the Hill coefficient constrained to ≤3 for both GABA and THIP to account for multiple binding sites. Comparisons of the sensitivity to agonist were made by the Extra sum-of-squares F test implemented in GraphPad Prism software with a p criterion of 0.05. Alternatively, individual cells were fit by the Hill equation, followed by a one-way ANOVA and Sidak’s multiple comparisons test if the ANOVA revealed a difference. For the latter approach, THIP data from 4 WT cells and 5 cells in the δT6’Y conditions were excluded due to non-converging fits or outlier EC_50_ values (>5 standard deviations beyond mean).

### Slice Preparation

Mice from WT, δT6’Y KI, and γ2T6’Y KI of either sex were used for experiments at postnatal day (P) 30 ± 2 days (Sun et al., 2018). Coronal brain slices at 300-μm thickness were cut in ice-cold, modified artificial CSF (aCSF containing in mM: 87 NaCl, 75 sucrose, 25 glucose, 25 NaHCO_3_, 2.5 KCl, 1.25 NaH_2_PO_4_, equilibrated with 95% oxygen-5% CO_2_ plus 0.5 CaCl_2_, 3 MgCl_2_; 320 mOsm). Slices were incubated in choline-based recovery aCSF (in mM: 92 choline chloride, 25 glucose, 30 NaHCO_3_, 2.5 KCl, 1.2 NaH2PO_4_, 20 HEPES, 2 thiourea, 5 Na ascorbate, 3 Na pyruvate, 2 CaCl_2_, and 1 MgCl_2_, oxygenated; 300 mOsm) at 32°C for 30 min, and then stored in regular aCSF (in mM: 125 NaCl, 25 glucose, 25 NaHCO_3_, 2.5 KCl, 1.25 NaH2PO_4_, oxygenated; 310 mOsm) for at least 1 h at 25°C before experimental recording. To measure GABA_A_R activation, 10 μM NBQX and 50 μM D-APV were added in the regular aCSF to inhibit ionotropic glutamate receptors.

### Slice Whole-Cell Recording

Brain slices were transferred into a recording chamber with oxygenated, regular aCSF perfused at 2 ml/min at 32°C. Hippocampal DGCs were identified by IR-DIC microscopy (Nikon FN1 microscope and Photometrics Prime camera). Whole-cell recordings were performed with pipettes pulled from borosilicate glass and with open tip resistance of 3–7 MΩ. Pipettes contained the following in mM: 130 CsCl, 10 HEPES, 5 EGTA, 2 MgATP, 0.5 NAGTP, and 4 QX-314; pH adjusted to 7.3 with CsOH; 290 (mOsm). Recordings began 5 min after whole-cell configuration was established. Signals were recorded using a MultiClamp 700B amplifier (Molecular Devices), Digidata 1550 16-bit A/D converter, and pClamp 10.4 software (Molecular Devices). To measure (4,5,6,7-tetrahydroisoxazolo[5,4-c]pyridin-3-ol) THIP currents, DGCs were voltage-clamped at −70 mV, and PTX (50 μM) was applied to block non δ-containing receptors in δT6’Y CRISPR KI slices, and the holding current was recorded throughout.

To measure the GABA sensitivity of δ-containing receptors, nucleated patches were obtained (Sather et al., 1992) to minimize the influence of GABA uptake on GABA responses. After PTX application, GABA at 1, 10, and 30 μM was applied. Data were plotted in Prism (Graphpad, San Diego, CA).

To measure subunit contributions to tonic current and to measure rectification of GABA_A_R-mediated current, DGCs from WT, δT6’Y KI, or γ2T6’Y KI slices were recorded at −70 mV. Exogenous GABA (5 μM) was applied to induce a basal tonic current, followed by PTX co-application (50 μM) to separate δ-containing receptors from γ2-containing receptors in the two KI lines. The competitive GABA_A_R antagonist gabazine (GBZ, 50 μM) was applied to block the remaining GABA_A_Rs at the end of recordings. Current remaining after the GBZ application was taken as a 0-level tonic current. For rectification, the current-voltage (*I*-*V*) relationship of tonic currents mediated by δ-containing receptors or γ2-containing receptors was measured by applying a slow (10 s) voltage ramp from −100 mV to 60 mV in the presence of Cd^2+^ to block voltage-gated Ca^2+^ channels. Rectification index (RI) represents the ratio of conductance of GABA_A_Rs at −60 mV and +60 mV and was calculated as follows: RI = [*I*_+__60_/(60 − *E*_rev_)]/[*I*_−60_/(−60 − *E*_rev_)].

### Drugs

GABA, pentobarbital, and salts were purchased from Sigma-Aldrich (St. Louis, MO). DS2 [4-chloro-N-2-(2-thienyl) imidazo[1,2-a]pyridine-3-yl benzamide] was purchased from R&D Systems (Minneapolis, MN). Steroids were from Sigma-Aldrich, or Steraloids (Newport, RI). Channel blockers NBQX and D-APV were from Tocris Bioscience (Minneapolis, MN).

## Results

### Agonist Sensitivity in Recombinant GABA_A_Rs and CRISPR Mutation Knock-In Mice

**Figure 1:**
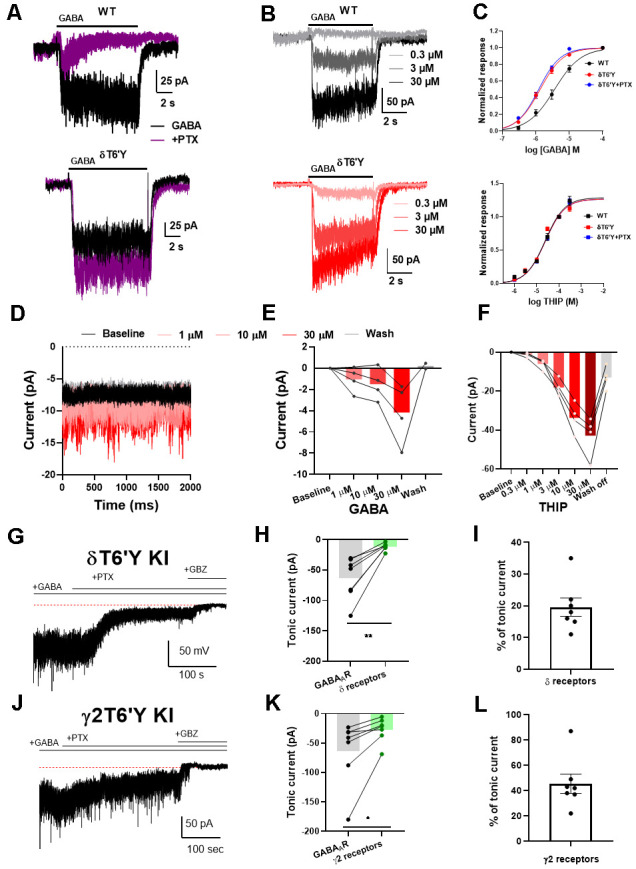
Agonist sensitivity in recombinant α4β2δ GABA_A_Rs expressed in N2a cells and KI mice. **(A)** Example traces of response to 1 μM GABA elicited from N2a cells transfected with wildtype (WT) GABA_A_R subunits. This response was abolished by co-application of 100 μM PTX. The δT6’Y mutation exhibited PTX-insensitivity when expressed with α4 and β2 subunits. **(B)** Representative traces of different GABA concentrations (0.3–30 μM) from WT GABA_A_Rs and δT6’Y mutation expressed in N2a cells. Shading indicates different GABA concentrations. **(C)** Concentration response curves for agonists GABA and THIP, respectively. For GABA, fits to the Hill equation yielded EC_50_ values of 3.61 μM, 1.33 μM, and 1.21 μM for WT and δT6’Y and δT6’Y in the presence of 50 μM PTX, respectively. The Hill coefficients were 1.1, 1.3, and 1.3 respectively. The EC_50_ values for WT and δT6’Y differed as assessed by the sum of squares F test (*p* < 0.0001). The sum of squares as a measure of goodness of fit was 0.386, 0.179, and 0.141 respectively for WT, δT6’Y, and δT6’Y + PTX. For THIP, fits to the Hill equation yielded EC_50_ values of 38 μM, 17 μM, and 31 μM for WT, δT6’Y, and δT6’Y + PTX, respectively with Hill coefficients of 0.79, 1.14, and 0.94. The p-value for a sum-of-squares F test on EC_50_ values of WT vs. δT6’Y was 0.061. The sum of squares as a measure of goodness of fit was 0.004, 0.010, 0.0007. **(D–F)** Slice recording from DGCs of δT6’Y KI mice showing agonist sensitivity of δ-containing GABA_A_Rs. All traces were obtained in the presence of 50 μM PTX. **(D)** Example traces of holding current after perfusing different concentrations of GABA. **(E)** Summary of current induced by different concentrations of GABA in nucleated patch-clamp recording (gray lines represent individual patches). **(F)** Summary of current induced by different concentrations of THIP in whole-cell recordings. **(G)** Representative whole-cell recording from δT6’Y KI DGC. Exogenous GABA (5 μM) induced a tonic current partially blocked by PTX (50 μM). Gabazine (GBZ) completely abolished the tonic current. **(H)** Summary of tonic currents mediated by δ-containing receptors and all GABA_A_R (*n* = 7, paired t-test, −63.6 ± 13.2 pA vs. −11.8 ± 2.2 pA, *p* = 0.0037). Both measurements are subtracted from the residual holding current in the presence of GBZ. **(I)** Percentage of tonic current mediated by δ-containing receptors (*n* = 7, 19.6% ±2.9%). **(J–L)** The same experiment from γ2T6’Y KI DGCs. Similar to δT6’Y KI DGCs, PTX partially blocked the GABA-induced tonic currents (*n* = 7, paired t-test, −63.2 ± 21.0 vs. −27.2 ± 7.9 pA, *p* = 0.0383). The contribution of γ2-containing receptors was calculated (*n* = 7, 45.4% ± 7.6), with the balance (~55%) mediated by non-γ2 containing receptors. PTX, picrotoxin; KI, knock-in; DGCs, dentate granule cells.

To characterize pharmacological and biophysical features of GABA_A_Rs containing the δT6’Y subunit, we first examined activation by the natural agonist GABA. Figure 1A shows a typical response elicited by 1 μM of GABA from wild type (WT) α4β2δ subunits transfected into N2a cells. The response was strongly inhibited when co-applied with the channel blocking antagonist PTX at 100 μM. PTX sensitivity was absent when δT6’Y was introduced with WT α4 and β2 subunits, but the activation by GABA was retained. Overall, there was no difference in the amplitude of GABA responses to 1 μM GABA in the presence or absence of 50 μM PTX (−129.7 ± 21.5 pA vs. −138.4 ± 22.2 pA, *n* = 27, 26; *p* = 0.78, *t-*test). We note that incorporation of δ subunits into recombinant receptors has been controversial, and PTX insensitivity of δT6’Y (or the comparable mutation in other subunits) yields a useful marker of subunit incorporation.

To further understand the impact of this mutation on the function of GABA_A_Rs, we investigated a full concentration-response relationship by varying GABA concentration in WT and δT6’Y receptors (Figures 1B,C). We observed that the EC_50_ of receptors containing the δ T6’Y mutation was left-shifted when compared to WT δ-containing receptors (1.33 μM and 3.61 μM, respectively, *p* < 0.001, Extra Sum of Squares F test). Alternatively, comparison of EC_50_ values by fits to data from individual cells by one-way ANOVA (see “Methods” section) showed similar results: overall effect on EC_50_ (*F*_(2, 21)_= 21.77; *p* < 0.001), with *post hoc* comparison revealing a difference between genotypes (adjusted p-value < 0.0001) but not between PTX conditions in the δT6’Y cells. However, we failed to detect a lower EC_50_ for the δ-preferring agonist THIP (Figure 1C lower panel 38.7 μM and 16.7 μM for WT and δ T6’Y mutated group, respectively; *p* = 0.06, Extra Sum of Squares F test). Individual fits for THIP revealed a similar result: (one-way ANOVA *F*_(2, 29)_ = 2.596, *p* = 0.092).

In addition, we performed GABA and THIP concentration-response experiments on receptors bearing the δT6’Y mutation in the presence of 50 μM PTX (Figure 1C, blue symbols). The EC_50_ for GABA but not THIP was detectably altered by the δT6’Y mutation, and PTX presence did not alter the EC_50_ on the mutated receptors (Figure 1). These values were very similar to those in the absence of PTX, further highlighting the lack of significant PTX effect on mutant receptor function.

The finding of reduced agonist EC_50_ values in recombinant GABA_A_Rs for the natural agonist GABA leads to the question of how agonists behave on δ GABA_A_Rs in native cells, especially since WT recombinant δ-containing receptors exhibit a range of estimated EC_50_ values, from low nM to high μM (Brown et al., 2002; Karim et al., 2012, 2013; Eaton et al., 2014; Wongsamitkul et al., 2016). Although we could not evaluate WT native δ GABA_A_Rs because of the lack of selective pharmacology, we evaluated δ-containing GABA_A_Rs using PTX-resistant δT6’Y KI DGCs, a cell type rich in δ-containing receptors. We performed nucleated patch recordings, lifted from the slice, to avoid the complication of GABA uptake, which lowers the local GABA concentration in tissue to indeterminant values (Isaacson et al., 1993). In the presence of PTX, exogenous GABA increased current in nucleated patches from 1 μM to 30 μM and recovered after GABA wash off (Figures 1D,E). The results suggest that the increased sensitivity to GABA evident in recombinant GABA_A_Rs is not readily evident in native cells. To examine receptor sensitivity using a method that doesn’t suffer from the disadvantages of patch excision and for which recombinant receptors did not exhibit clear alteration in sensitivity, we examined whole-cell current activated by the poorly transported δ-preferring agonist THIP. Similar to GABA application, the current mediated by δ-containing receptors increased as THIP concentration increased from 1 μM to 30 μM (Figure 1F). These results show that in DGCs the EC_50_ for both natural and δ-preferring agonists is in the micromolar range rather than in the nM range as has been observed in some recombinant receptor experiments.

Given that the response of δT6’Y-containing receptors in native cells does not appear to exhibit the higher sensitivity to GABA observed in recombinant receptors, we evaluated the contribution of δ-containing receptors to GABA-induced tonic current using both δT6’Y and γ2T6’Y knock-in mouse lines (Sun et al., 2018; Lu et al., 2020). In mouse hippocampal DGCs, we induced tonic current with 5 μM GABA, typical of previous work (Lee and Maguire, 2014). In both δT6’Y KI and γ2T6’Y KI DGCs, 5 μM GABA induced ~60 pA tonic current. This current was partially blocked by 50 μM PTX, leaving PTX-resistant tonic current. GBZ (50 μM) was applied to eliminate the remaining tonic current (Figures 1G,J). Surprisingly, δ-containing receptors mediated ~20% of the GABA-induced tonic current (Figures 1H,I), judged by PTX-resistant current in δT6’Y KI cells. By contrast, non-γ2 containing receptors mediated 55% of the tonic current from the γ2T6’Y cells (Figures 1K,I). Both mouse lines indicate a smaller contribution of δ-containing receptors to GABA-induced tonic current than previous work has suggested. Possible reasons for the discrepancy in estimates for δ contribution within the two KI mouse lines are evaluated in the Discussion.

### Rectification Does Not Differ Among Native GABA_A_R Isoforms

**Figure 2:**
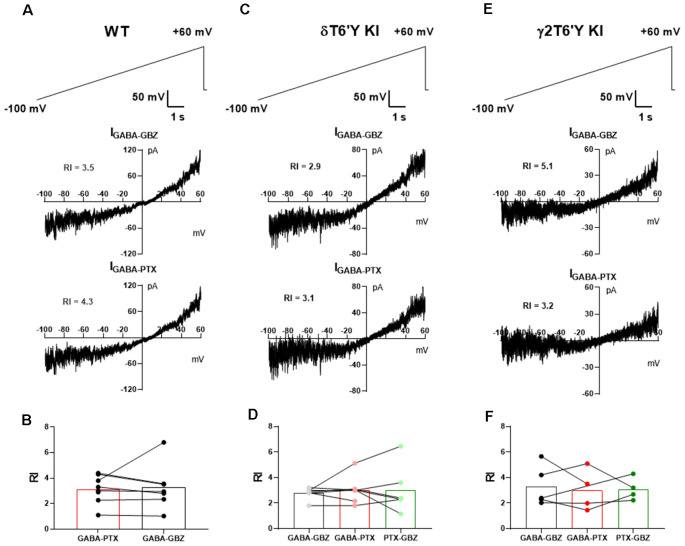
Rectification does not differ among GABA_A_R isoforms in slice recording. **(A)** In a representative WT DGC, GABA_A_R-mediated tonic current shows outward rectification. **(B)** Summary of GABA_A_R rectification index (RI). PTX and GBZ show similar blockage of GABA_A_R-mediated tonic current in WT DGCs (*n* = 7, paired-*t* test, 3.1 ± 0.4 vs. 3.3 ± 0.7, *p* = 0.8275). **(C,D)** Tonic current mediated by δ-containing GABA_A_Rs shows outward rectification. Note that RI of total GABA_A_Rs, non δ-containing GABA_A_Rs, and δ-containing GABA_A_Rs are shown as GABA-GBZ, GABA-PTX, and PTX-GBZ, respectively. One-way ANOVA showed no drug effect on RI (*F*_(1.195, 6.575)_ = 0.1512, *p* = 0.7532). **(E,F)** Tonic current mediated by γ2-containing GABA_A_Rs shows outward rectification. One-way ANOVA showed no drug effect on RI (*F*_(1.587, 4.762)_ = 0.1155, *p* = 0.8518). RI values in the various conditions were obtained from antagonist subtractions as indicated below the graphs in **(B,D,F)**.

We extended the examination of native receptors in DGCs to rectification properties. GABA_A_R-mediated tonic current shows strong outward rectification in CA1 pyramidal cells (Pavlov et al., 2009). Outward rectification of conductance in the range of voltages from −70 mV to near action potential threshold will influence excitability, and disproportionate outward rectification of GABA current mediated by δ-containing receptors could perhaps help explain the small contribution of these receptors in our experiments compared with previous work (Stell et al., 2003; Wei et al., 2003). To examine whether outward rectification is stronger in δ-containing GABA_A_Rs than in γ2-containing receptors, we measured the tonic current of DGCs while applying a slow voltage ramp from −100 mV to +60 mV (Figures 2A–E). After applying sequential PTX and GBZ in slices from PTX-resistant δT6’Y KI and γ2T6’Y KI mice, we calculated the rectification index of GABA current mediated by δ-containing receptors and by γ2-containing receptors, respectively (Figures 2A–E). Our results suggest that rectification is similar among the three genotypes (Figures 2B,D,F).

### Positive Allosteric Modulator (PAM) Sensitivity in Recombinant Receptors

**Figure 3:**
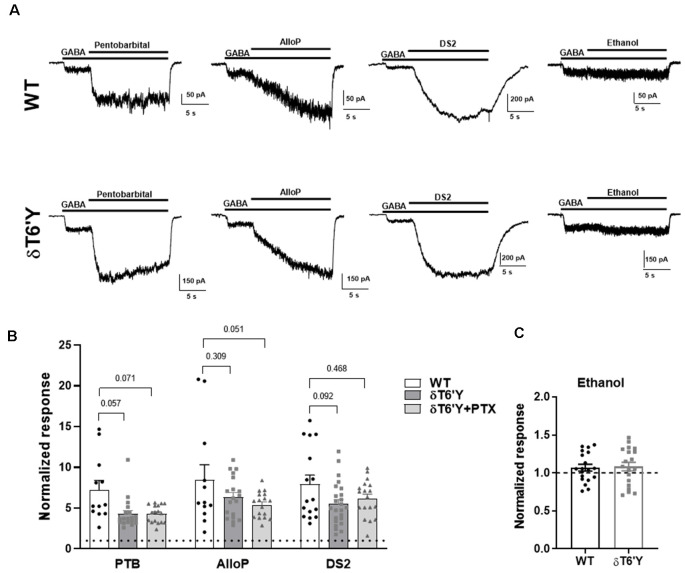
PAM sensitivity in recombinant α4β2δ GABA_A_Rs expressed in N2a cells. **(A)** Example traces of response elicited from GABA (1 μM) potentiated by pentobarbital (25 μM) and AlloP (50 nM) in WT GABA_A_Rs, δT6’Y GABA_A_Rs as indicated. **(B)** Summary of pentobarbital, AlloP and, DS2 effects on GABA current with 10 s application time (*n* = 12–19) in WT δ and δT6’Y containing receptors. Note that δT6’Y in the presence of 50 μM PTX is also represented. A two-way ANOVA showed a main effect of genotype (*F*_(2, 145)_ = 10.04, *p* < 0.0001), with p-values for *post hoc* Sidak’s multiple comparison test given above the graphs. **(C)** Ethanol sensitivity was evident as a slight increase above baseline in WT or δT6’Y GABA_A_Rs (two-way ANOVA (*F*_(1, 36)_ = 4.497; *p* = 0.04, *n* = 19 per group). However, no genotype or genotype by ethanol interaction was evident (interaction: *F*_(1, 36)_ = 0.038; *p* = 0.85). PAM, Positive Allosteric Modulator.

Many important experimental and clinically used compounds augment GABA_A_R function. Do these compounds act as expected at receptors containing the δT6’Y mutation? We tested pentobarbital, a barbiturate; allopregnanolone (AlloP), an endogenous neurosteroid; DS2, a δ-receptor preferring positive allosteric modulator (PAM; Jensen et al., 2013); and ethanol. Figure 3 shows that pentobarbital (50 μM), AlloP (50 nM), and DS2 (1 μM) all potentiated responses to 1 μM GABA in both WT and δT6’Y receptors (Figures 3A,B). A two-way ANOVA applied to data in Figure 2B revealed that overall, δT6’Y exhibited less potentiation from modulators (*F*_(2, 145)_ = 10.04, *p* < 0.001. However, *post hoc* comparisons showed only marginal differences for each modulator (Figure 3B). Ethanol modestly potentiated both WT and δT6’Y currents at 30–100 mM (Figure 3C; 100 mM shown). We attribute the overall reduced sensitivity of δT6’Y receptors to positive modulators to be the likely secondary consequence of reduced agonist EC_50_ (Figure 1). Overall, we conclude that positive allosteric modulators act similarly in WT and δT6’Y-containing receptors.

### DS2-Sensitive PTX-Insensitive Responses in Rat Hippocampal Cultures

**Figure 4:**
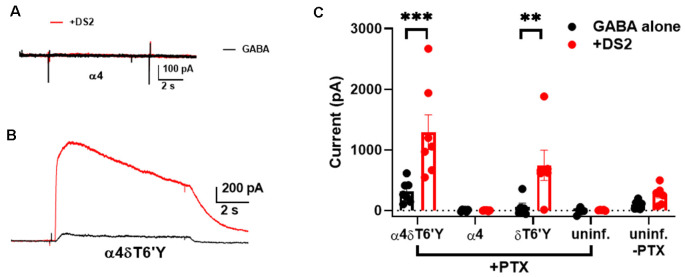
Viral overexpression of δT6’Y mutation, along with WT α4 subunit, yields DS2-sensitive, PTX-insensitive GABA current in rat hippocampal cultures. All data were collected in the presence of 50 μM PTX except for the far pair of bars in panel **(C)**. **(A,B)** Representative responses to 0.2 μM GABA from a neuron transduced with α4 subunit and from a neuron transduced with both α4 and δT6’Y virus as indicated. The α4 virus alone condition demonstrates that there is no breakthrough GABA current in the presence of PTX. DS2 (1 μM) enhanced the GABA response in δT6’Y-containing receptors, indicating functional δT6’Y expression. **(C)** Summary of GABA currents following indicated viral transduction. Colors illustrate GABA (in black) application and co-application with 1 μM DS2 (in red). The weak DS2 effect in non-transduced cultures without PTX indicates low δ expression at baseline. **p < 0.01, ***p < 0.001.

Hippocampal neurons prominently express γ2-containing GABA_A_Rs that traffic mainly to synapses, while δ-containing receptors are located perisynaptically or extrasynaptically, mainly in DGCs in the hippocampus (Wei et al., 2003). Because δ-containing receptors are thought to mediate mainly tonic GABA currents, we tested whether δ receptors are excluded from activation by synaptic GABA release. We used viral transduction of δT6’Y along with the preferred δ partner α4 in hippocampal neurons. GFP + α4 and non-transduced cells were used as controls. First, we evaluated the functional expression of transduced subunits. We used PTX resistance and sensitivity to the δ-preferring PAM DS2 to implicate δ-containing receptors. Figures 4A,B shows representative responses to 0.2 μM GABA ± 1 μM DS2 in the presence of PTX in control and δT6’Y neurons. WT cells demonstrated that there was little “breakthrough” GABA current in the presence of PTX. These examples and summary data in Figure 4C in the absence of PTX suggest that control cultures have almost no endogenous δ in functional receptors, probably owing to the prominence of pyramidal neurons rather than granule neurons in the cultures. δT6’Y alone (without α4) was also capable of sustaining responses to GABA and DS2, suggesting that δT6’Y can partner with endogenous α and β subunits to form functional receptors (Figure 4C).

### Synaptic Activation of Overexpression of δT6’Y Mutation in Rat Hippocampal Cultures

**Figure 5:**
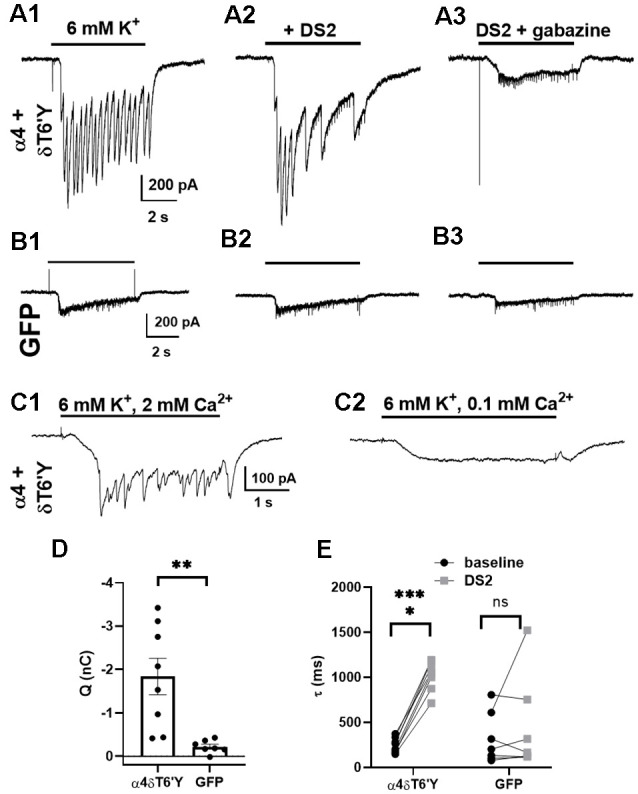
Synaptic activation of transduced δT6’Y subunits in rat hippocampal cultures. **(A1–3)** Example traces of IPSCs induced by 6 mM K^+^ depolarizing stimulation after transduction of δT6’Y subunits along with α4 subunits **(A1)**. NBQX and D-APV were used to block AMPAR and NMDAR activation, respectively, and 50 μM PTX was applied throughout. The K^+^ evoked IPSCs were enhanced by co-application with 1 μM DS2 **(A2)**, and sensitive to 10 μM gabazine **(A3)**. The frequency of IPSCs may be reduced in **(A2)** due to enhanced network inhibition with DS2 application. Residual transient inward currents may represent small breakthrough IPSCs or small action currents elicited by K^+^ depolarization in poorly clamped dendrites. **(B1–3)** The same protocol was applied to neurons transfected with GFP alone. The current initiated by 6 mM K^+^ was smaller, and DS2 enhancement was absent. **(C1,2)** In the presence of PTX, NBQX, and D-APV, phasic events in a δT6’Y + WT α4 transduced neuron were blocked by reduced Ca^2+^ (*n* = 10/10 cells), consistent with the synaptic release of GABA driving postsynaptic responses. Upon reduction of extracellular Ca^2+^ to 0.1 mM in panel **(C2)**, extracellular Mg^2+^ concentration was increased to 3 mM to preserve total divalent cation concentration. **(D)** Quantification of PTX-insensitive synaptic charge for δT6’Y and control transfections (*n* = 8, 7, unpaired t-test with Welch’s correction, *p* = 0.006). The charge was integrated over the duration of K^+^ application, and charge in the presence of GBZ was subtracted for the plots. **(E)** Summary of decay time constant τ (ms) of the final IPSC of the barrage caused by K^+^ application. DS2 significantly prolonged the decay of synaptic currents, indicating δ contribution to the α4 + δT6’Y group but not the GFP control cells (*n* = 8, 7; 2-way ANOVA with Sidak’s multiple comparison test *P* < 0.0001 for α4 + δT6’Y). The additional variability in **(D)** for the GFP control data likely arises from the fits to small-amplitude currents [e.g., **(B1)** and **(B2)** vs. **(A1)** and **(A2)**]. *p < 0.05, **p < 0.01, ***p < 0.001, ns = not significant.

Given the robust expression of δT6’Y in hippocampal cultures (Figure 4), we explored whether δ receptors are accessed by the synaptic release of GABA. We recorded from infected cells while challenging the surrounding network with 6 mM potassium in the presence of PTX and the glutamate receptor blockers NBQX and D-APV. The currents in Figure 5A thus represent PTX-insensitive IPSCs from the recorded cell. The contribution of δ receptors was verified with DS2 (1 μM) application, which prolonged IPSCs (Figures 5A,E). IPSCs were also sensitive to GBZ, a competitive GABA_A_R antagonist (Figure 5A3). In control GFP-only cells (also recorded in the presence of receptor antagonists), potassium-elicited currents were much smaller (Figures 5B,D), reflecting primarily direct potassium effects, and were insensitive to DS2 and to GBZ (Figures 5B1–B3,E). To test that phasic events were indeed synaptically driven, we confirmed that lowering bath Ca^2+^ eliminated the phasic events in each of the 10 cells tested (Figure 5C). We conclude that δ subunit-containing receptors are readily activated by synaptically released GABA, at least under conditions of exogenous transduction. Note that our experiments do not determine whether the activated receptors are anatomically synaptic, perisynaptic, or extrasynaptic.

## Discussion

We have evaluated the effect on the receptor function of a point mutation in the δ subunit of GABA_A_Rs expressed heterologously and as a knock-in in DGCs. The point mutation has the intended effect on PTX sensitivity. However, the results also show that the mutation is not completely silent in the absence of PTX. The primary change in receptor function is a reduction in GABA EC_50_ in recombinant GABA_A_Rs, which may also account for the reduced sensitivity of receptors to positive allosteric modulators at a fixed agonist concentration. EC_50_ differences could arise from genuine changes in the binding properties of the receptor or from changes to the gating properties of the receptor (Colquhoun, 1998). Given that the amino acid substitution occurs within TM2, near important pore-forming residues, it seems likely that gating properties are primarily affected and agonist binding affected indirectly.

A puzzling attribute of the mutant receptors is the altered EC_50_ for GABA but not for THIP (Figure 1). Although speculative, a possible reason for the difference is the partial agonist quality of GABA compared with the full agonist properties of THIP at δ subunit-containing receptors (Brown et al., 2002). The mutation may affect the EC_50_ for partial agonists but not for full agonists, because of different receptor conformations adopted by bound full vs. partial agonists.

We previously used δT6’Y mice, also employed in the current study, along with γ2T6’Y knock-in mice to argue that δ receptors contribute more to synaptic IPSCs in DGCs than prevailing views typically suggest (Sun et al., 2018, 2020). Our present experiments reinforce these observations with an acute expression of δ subunits in primary cultures (Figures 4, 5). Several pieces of evidence suggest that the phasic events are of synaptic (vesicular) origin rather than from reverse GABA uptake. First, the events are Ca^2+^ dependent (Figure 5C). Although interactions between synaptic glutamate release and reverse GABA transport are formally possible (Guimarães-Souza et al., 2011), reverse GABA transport is evoked by stronger depolarization and with a slower time course than in our experiments (Gaspary et al., 1998; Allen et al., 2004). Note that neither our previous experiments nor the present experiments speak to the anatomical location of the δ containing receptors. Rather, the results suggest that δ receptors can be accessed by synaptic GABA release and contradict the view that δ receptors solely mediate tonic current. Previously, using PTX-resistant γ2 knock-ins, we used PTX isolation and digital subtraction to isolate WT δ IPSCs in DGCs and failed to discern a difference in amplitude or decay kinetics of δ mediated IPSCs, between WT and δT6’Y IPSCs (Sun et al., 2018, 2020), based on the assumption that δ receptors in γ2T6’Y mice retain WT features. Therefore, it appears that the mutation has a limited functional impact on DGCs. The possible reasons are considered below.

The lower EC_50_ to GABA of δT6’Y receptors predicts a larger tonic current in T6’Y DGCs in response to a fixed, low GABA concentration, but this prediction was not borne out. We also previously showed that responses to a sub-saturating concentration of the δ-preferring agonist THIP were not discernibly different from those of WT receptors in DGCs (Sun et al., 2018; Lu et al., 2020). If tonic GABA responses were augmented because of the lower agonist EC_50_ in T6’Y receptors, we may have overestimated δ contribution, exacerbating the contrast with prevailing views. Here we also examined a range of agonist concentrations in δT6’Y KI cells to test a hypothesis of exceptionally high-affinity delta receptors (Karim et al., 2012; Eaton et al., 2014). Our study constitutes the first direct assessment of GABA sensitivity of δ receptors in native cells and suggests that the EC_50_ of DGC δ receptors is in the micromolar range, even with a possible three-fold lowering induced by mutation. Thus, at least in DGCs, GABA and THIP are not particularly high-potency agonists at native δ receptors. Although the EC_50_ values differ from some published values (Karim et al., 2012; Eaton et al., 2014; Pan et al., 2018), our observations are in line with others (You and Dunn, 2007; Mortensen et al., 2010). Our results in fact appear consistent with a particular subunit arrangement in oocytes, forced by concatemerized subunits (Eaton et al., 2014).

Our results show that δ-containing receptors mediate ~20% of GABA_A_R tonic current in δT6’Y KI DGCs, while in γ2T6’Y KI DGCs, non γ2-containing receptors mediate ~55% of the tonic current. Although both are lower than previous estimates, a possible reason for the internal discrepancy is that non-γ2 receptors besides δ receptors (e.g., γ1-containing receptors and αβ-containing receptors) contribute to PTX-sensitive current in γ2T6’Y cells (Mortensen and Smart, 2006). The lower estimate of contribution from δ receptors in δT6’Y cells suggests that high agonist affinity of δT6’Y (Figure 1C) does not dominate, consistent with direct studies of GABA EC_50_ in DGCs in Figures 1D,E. Poor functional expression of δT6’Y receptors might explain the low δ contribution to GABA tonic current in δT6’Y cells, but previously we presented multiple lines of evidence that δT6’Y expression is near that of WT in DGCs (Sun et al., 2018). Further, in our current work the total GBZ-sensitive GABA current amplitude in the two mouse lines did not differ (Figures 1H,K). Thus, the evidence seems to favor non-γ2/non-δ receptor contributions to the tonic current to explain the discrepancy in Figure 1I vs. Figure 1L.

Positive allosteric modulator sensitivity is important since many drugs of this class are in clinical use or in clinical development. Subunit selective modulators could represent a new generation of therapeutics (Rudolph and Möhler, 2006, 2014). For instance, neurosteroids may have persistent antidepressant effects by virtue of selectivity for δ receptors (Maguire and Mody, 2008; Melón et al., 2018; Meltzer-Brody and Kanes, 2020). On the other hand, our recent studies suggested that δ receptors in the presence or absence of GABA or THIP seem to exhibit little if any neurosteroid selectivity over γ2 receptors (Lu et al., 2020). Here we found that recombinant δT6’Y receptors retained sensitivity to allosteric modulators, including AlloP and ethanol, with both WT and δT6’Y receptors exhibiting very limited sensitivity to ethanol in our hands. This finding differs from some previous results (Wallner et al., 2006) but not others (Borghese et al., 2006). Although an analysis of variance showed an effect of the δT6’Y mutation on positive allosteric modulation, *post hoc* analyses of individual PAMs indicated this global effect was largely accounted for by reduced efficacy of PTB (Figure 3). Because very different classes of compounds were used in these experiments and no one compound accounted entirely for the reduced overall effect (Figure 3), we hypothesize that the change in agonist sensitivity ultimately underlies the difference in the effects of modulators at a fixed agonist concentration. The increased effectiveness of a fixed agonist concentration at mutated receptors yields a smaller dynamic range for PAMs. In our previous studies, this effect could have caused us to underestimate AlloP potentiation in native cells bearing the δT6’Y mutation. However, complementary results in native cells bearing γ2T6’Y suggest that our conclusions in native cells about AlloP selectivity are not affected by this property of δT6’Y receptors (Lu et al., 2020). In addition, results in Figure 1E suggest that GABA sensitivity in native cells may not be altered as they are in recombinant receptors (Figure 1C).

There are several reasons that differences in agonist sensitivity observed in recombinant receptors might not be evident in DGC native receptors. We cannot completely exclude the possibility that native cells exhibit the same three-fold higher sensitivity for GABA observed in recombinant receptors; experiments in native cells embedded in tissue slices may lack the precision obtainable in cultures, where exposure to solution and drug is highly controlled. On the other hand, there are potential reasons that a change observed in recombinant receptors expressed in heterologous cells may not be evident in native receptors. One possibility is that the beta subunit differs in our recombinant receptors vs. native cells, which could alter channel behavior. Another possibility is that post-translational modification or interacting proteins differ in the two environments. Regardless, the caveat of agonist potency will be important to bear in mind when studying other cell types.

GABA_A_Rs have voltage sensitivity that has been previously suggested to contribute to inhibition by tonic current (Weiss, 1988; Pavlov et al., 2009). However, to our knowledge rectification has been examined in recombinant δ GABA_A_Rs (Brown et al., 2002) but not native δ-containing GABA_A_Rs. We used δT6’Y to explore the hypothesis that δ receptors may exhibit more rectification than γ2 receptors, giving rise to a more prominent δ receptor contribution to tonic inhibition than our previous results at a fixed membrane potential have suggested. In contrast to the possibility of disproportionate δ receptor rectification, we found no difference in rectification properties among receptor subclasses in DGCs, so stronger rectification is unlikely to contribute to a more prominent role of δ receptors in inhibition.

In summary, our results suggest that δT6’Y is a helpful tool for exploring the role of GABA_A_R subpopulations in neuronal physiology and pharmacology. Caveats related to altered agonist sensitivity have not been detectable in KI mice. Differences detected in recombinant receptors need to be considered when interpreting data from knock-in mice. However, with regard to previous results with DGCs, we see no reason for conclusions drawn to date to be revised. If the changes to baseline function are detected in other native cell types, the present results may help to circumvent the limitations.

## Data Availability Statement

The raw data supporting the conclusions of this article will be made available by the authors, without undue reservation.

## Ethics Statement

The animal study was reviewed and approved by Washington University IACUC. Written informed consent was obtained from the owners for the participation of their animals in this study.

## Author Contributions

SM, JB, JS, and CZ designed research. H-JS and XL performed research. H-JS, XL, JS, CZ, and SM wrote the article. All authors contributed to the article and approved the submitted version.

## Conflict of Interest

CZ serves on the Scientific Advisory Board to Sage Therapeutics. The remaining authors declare that the research was conducted in the absence of any commercial or financial relationships that could be construed as a potential conflict of interest.

## Publisher’s Note

All claims expressed in this article are solely those of the authors and do not necessarily represent those of their affiliated organizations, or those of the publisher, the editors and the reviewers. Any product that may be evaluated in this article, or claim that may be made by its manufacturer, is not guaranteed or endorsed by the publisher.
